# Total and Free 25-Hydroxy-Vitamin D and Bacterial Vaginosis in Pregnant African American Women

**DOI:** 10.1155/2019/9426795

**Published:** 2019-01-01

**Authors:** Anne L. Dunlop, Sheila L. Jordan, Erin P. Ferranti, Cherie C. Hill, Shiven Patel, Li Hao, Elizabeth J. Corwin, Vin Tangpricha

**Affiliations:** ^1^Nell Hodgson Woodruff School of Nursing, Emory University, Atlanta, GA 30322, USA; ^2^Department of Family & Preventive Medicine, Emory University School of Medicine, Atlanta, GA 30322, USA; ^3^Department of Obstetrics & Gynecology, Emory University School of Medicine, Atlanta, GA 30322, USA; ^4^Division of Endocrinology, Metabolism & Lipids, Department of Medicine, Emory University School of Medicine, Atlanta, GA 30322, USA; ^5^Atlanta VA Medical Center, Atlanta, GA 30033, USA

## Abstract

**Objective:**

This study sought to investigate associations between serum total and free 25(OH)D and bacterial vaginosis (BV) in early and later pregnancy among US black women to provide insight into the most clinically relevant measure of vitamin D status among pregnant black women with respect to risk for BV as well as insights into critical time points for measuring and/or addressing vitamin D status in pregnancy.

**Methods:**

Data and biospecimens were derived from a subsample (N = 137) of women from the Emory University African American Vaginal, Oral, and Gut Microbiome in Pregnancy Cohort, for whom data related to vitamin D status (serum assays for total and free 25(OH)D) and Nugent score of Gram stained vaginal specimens in early (8-14 weeks) and later (24-30 weeks) were available. We compared total and free 25(OH)D concentrations for women according to Nugent score category (normal flora, intermediate flora, and BV) and assessed the odds of BV according to measures of vitamin D status.

**Results:**

Thirty-seven (27%) women had adequate vitamin D status at baseline, whereas 70 (51%) had insufficient vitamin D and 30 (22%) were vitamin D deficient; there were not significant differences in the proportion of women with adequate, insufficient, or deficient vitamin D according to Nugent score category. However, the odds of BV later in pregnancy were significantly higher for women who experienced a smaller rise in total 25(OH)D and free 25(OH)D from 8-14 through 24-30 weeks gestation.

**Conclusion:**

The change in measures of vitamin D status from early to later pregnancy is associated with the occurrence of BV in pregnancy. Further research is needed to examine the association between the change in vitamin D status over pregnancy and the occurrence of BV and other measures of vaginal microbial composition as well as to identify factors that influence change in vitamin D status over pregnancy.

## 1. Introduction

Bacterial vaginosis (BV) is a polymicrobial vaginal condition characterized by a reduction in acid-producing* Lactobacilli* and overgrowth of anaerobic Gram-negative and Gram-variable bacteria, including* Mycoplasma hominis, Bacteroides, Mobiluncus,* and* Garnerella vaginalis* [1]. Women with BV are at higher risk for acquiring and transmitting sexually transmitted infections [2–4], and pregnant women with BV are at increased risk for premature rupture of membranes [5], chorioamnionitis [6], spontaneous preterm birth [7], and post-cesarean endometritis [8]. RCTs among women with a previous spontaneous preterm birth support a reduction in preterm birth recurrence following early screening and treatment for BV [9].

Black women are more commonly affected by BV, with prevalence estimates of 51.4% for US black women compared to 23.2% for US white women of reproductive age [10]. The significantly higher rates of BV among black women are estimated to account for up to one-third of the US black-white racial disparity in preterm birth [11, 12]. While douching, number of sexual partners, and lower socioeconomic status increase a woman's risk for BV, these risk factors do not account for the disparity in BV rates [11, 13]. Factors that impair the immune system, such as micronutrient deficiencies, may also increase susceptibility to BV. Deficiency of vitamin D influences a number of aspects of the immune system [14] and has been linked to BV [15, 16]. Studies of US women of reproductive age indicate that approximately 80% of US black women are vitamin D deficient, compared with 13% of US white women [17, 18]. Black women's excess rate of vitamin D deficiency is attributed to darker skin pigmentation, which reduces cutaneous synthesis of cholecalciferol from exposure to sunlight, and lower intake through dietary and supplement sources [19, 20].

The concentration of total 25-hydroxyvitamin D [25(OH)D] is considered the barometer for vitamin D status [21]. Interestingly, multiple studies have found that despite blacks' lower total 25(OH)D compared to whites, increased disease risk is not consistently observed in blacks, suggesting that total 25(OH)D may not be the biomarker of vitamin D status most related to health effects [19]. The recent literature suggests that free 25(OH)D, which circulates unbound to vitamin D binding protein (VDBP) or albumin, may be a better marker of vitamin D bioavailability and function than total 25(OH)D [22], especially in racial/ethnic minority populations and/or during pregnancy due to varying affinity and levels of VDBP [23, 24].

Until recently, the concentration of free 25(OH)D could only be estimated using formula that consider levels of total 25(OH)D, VDBP, and albumin, but assays for measuring free 25(OH)D have since been developed [22]. Stronger associations with free 25(OH)D than with total 25(OH)D have been reported for serum calcium and parathyroid hormone [25], bone mineral density [26], and vascular outcomes [27], suggesting that free 25(OH)D may be a more clinically relevant measure. However, other studies have not found stronger associations with free compared to total 25(OH)D [28]. To date, no reports in the literature have compared the associations between directly measured free 25(OH)D and total 25(OH)D and health effects during pregnancy. The affinity of VDBP for vitamin D metabolites is lower during pregnancy, resulting in higher levels of free relative to total 25(OH)D), which is not accounted for in the mathematical formula [29].

This study sought to investigate associations between concentrations of total and free 25(OH)D and BV in early and later pregnancy among US black women. Findings from this study may provide guidance about the most clinically relevant measure of vitamin D status among pregnant black women with respect to risk for BV as well as insights into critical time points for measuring and/or addressing vitamin D status in pregnancy.

## 2. Materials and Methods

### 2.1. Overview

Participants for this study were drawn from women enrolled in the Emory University African American Vaginal, Oral, and Gut Microbiome in Pregnancy Cohort [30] for which pregnant US-born black women are recruited from two hospitals in Atlanta, GA: a private hospital affiliated with Emory University that provides services for a socioeconomically and educationally diverse population and a county-supported hospital that provides services to the low-income and underserved. Women presenting to the hospital-affiliated clinics for a prenatal care between 8 and 14 14 weeks gestation, as determined by standard criteria based upon last menstrual period and/or first trimester ultrasound, and who self-identified as African American, were offered cohort enrollment if they met the following inclusion criteria: (1) US-born black by self-report; (2) between 8 and 14 weeks gestation with a singleton pregnancy (verified by clinical record); (3) Able to comprehend written and spoken English; (4) between 18 and 40 years of age; (5) experiencing no chronic medical condition or taking prescribed chronic medications (verified by clinical record). The present study involved the comparison of total and free 25(OH)D concentrations for enrolled women with and without BV based on Nugent's criteria of Gram stained vaginal samples obtained concurrent with serum samples for 25(OH)D measurement at 8-14 weeks and 24-30 weeks gestation. This study was approved by the Emory University Institutional Review Board; all participants provided written informed consent for participation.

### 2.2. Study Sample

Women selected for inclusion in this study (N = 137) are a subsample of the women enrolled into the Emory University African American Vaginal, Oral, and Gut Microbiome in Pregnancy Cohort between April 1, 2014, and October 31, 2015, for whom both biochemical and clinical data related to vitamin D and BV status were available. Of the 184 women who enrolled in the cohort during that time period, 137 had complete data available for serum 25(OH)D measurement and vaginal Gram staining for BV determination at both data collection time points; the 47 women excluded from the present analysis had only data from the initial but not the subsequent time point available due to not completing the second study visit because of moving out of the area or discontinuing prenatal care at the clinical site.

### 2.3. Data Collection

Data collection for this study took place at prenatal care clinics during routine clinical appointments at two time points (Visit 1, between 8 and 14 weeks gestation; Visit 2, between 24 and 30 weeks gestation) and via medical record abstraction at the conclusion of the pregnancy. Complete data collection for the cohort is described in detail [30]. Measures of relevance to this study are presented below.

#### 2.3.1. Clinical and Questionnaire Data

*(1) Maternal race* was based on self-report of racial identity;* (2) Maternal age and prenatal health insurance status (Medicaid, private) *at the time of enrollment were ascertained from the prenatal record; in Georgia, women qualify for Medicaid in pregnancy if their household income is less than 200% of the federal poverty level;* (3) Gestational age* at the time of the study visits was ascertained from the prenatal record; all participants had early pregnancy dating by last menstrual period and/or early ultrasound, given enrollment criteria;* (4) Body mass index (BMI)* was calculated from patients' measured height and weight at the first prenatal visit ascertained from the prenatal record; (5)* Receipt of antibiotics in the month before or between study visits *was determined by abstraction of the prenatal record to ascertain whether the prenatal care provider prescribed or administered antibiotics (oral, vaginal, or parenteral) for any clinical diagnosis. The presence of clinical diagnoses was ascertained via clinical abstraction of the prenatal record, and involved ascertaining whether the clinician had diagnosed bacterial vaginosis on the basis of Amsel criteria [31], sexually transmitted infections (chlamydia, gonorrhoea, and trichomoniasis) on the basis of clinical laboratory testing results, urinary tract infection on the basis of urine culture, or any other bacterial infection (e.g., cellulitis, sinusitis) as well as the gestational age for which antibiotics were prescribed for the infection in comparison to the gestational age of the study visits for determination of antibiotic administration before or between study visits.

#### 2.3.2. Biological Specimens and Assays

At both prenatal study visits, vaginal swabs and venous blood samples were obtained from participants as part of the research study [30]; the results of research-related tests were not part of the clinical prenatal record and were considered in the prenatal care provided to participating women.

*(1) Vaginal Swab Collection for Nugent Scoring and Assignment of BV Status*. Study coordinators used verbal and pictorial instruction to instruct participating women in the self-collection of mid-vaginal swabs, which were immediately transported to the Emory Clinical Microbiology Laboratory for Gram staining according to Nugent's criteria. Nugent scores were classified into three levels: normal flora, score < 4; intermediate flora, score between 4 and 6; and BV, score ≥ 7 [32]. Nugent scoring was done via a single assessor who was blinded to participant identification and all clinical details, including symptoms and clinical diagnoses.

*(2) Quantification of Serum 25(OH)D*. Aliquots of maternal serum obtained from the venous blood draw were stored at -80°C until assayed for total and free 25(OH)D at the Emory University School of Medicine Vitamin D Research Laboratory, which participates in the vitamin D external quality assessment scheme (http://www.deqas.org/) and the National Institutes of Health/National Institute of Standards and Technology Vitamin D Metabolites Quality Assurance Program (VitDQAP). Total 25(OH)D was assayed the iSYS automated chemiluminescent assay (Immunodiagnostic Systems, Fountain Hills, AZ) with a range of detection of 2-120 ng/mL. Definitions of vitamin D status were based on the Endocrine Society prescribed reference ranges for vitamin D deficiency, total 25(OH)D < 20 ng/mL; vitamin D insufficiency, total 25(OH)D between 20 and 30 ng/mL; and vitamin D adequate, total 25(OH)D > 30 ng/mL [33]. Free 25(OH)D was measured directly with a competitive ELISA, calibrated against a symmetrical dialysis method (DIAsource ImmunoAssays, Louvain-la-Neuve, Belgium), with a range of detection of 2.4 – 17.1 pg/mL.

### 2.4. Data Analysis

We began analysis by assessing for missing values and checking statistical assumptions. Measures of total 25(OH)D, free 25(OH)D and Nugent score were found to be non-normally distributed. Descriptive statistics were used to evaluate differences in the characteristics of pregnant women by Nugent score category (normal flora, intermediate flora, BV) using Chi-square test, or Fisher's exact test in the case of small sample sizes, for categorical variables and one-way analysis of various (ANOVA) for continuous variables. In paired analysis, we compared the median total and free 25(OH)D from Visit 1 to Visit 2 using the Wilcoxon Signed Rank test. We also used the Chi-square test to compare the proportion of women with and without BV according to vitamin D status (categorized as adequate, insufficient, or deficient). We evaluated correlations between total and free 25(OH) concentrations and Nugent score using Spearman's correlation coefficient and used the Kruskal-Wallis ANOVA to compare the distribution of total and free 25(OH)D concentrations for women by Nugent score category. We used multinomial logistic regression models to evaluate associations between the outcome of Nugent score category (categorized as normal flora, intermediate flora, or BV) at the study visits and total and/or free 25(OH)D concentrations as the primary predictors, adjusting for relevant co-variates previously identified in the literature, including age, parity, first prenatal BMI, gestational age of sample, and receipt of antibiotics [15, 16, 34], which were forced entry terms into the model. A significance level of .05 was employed for all statistical testing. We evaluated whether adjusting for co-variates significantly improved model fit compared to an intercept only model using likelihood-ratio chi-square tests for goodness of fit of the model. We used this same multinomial logistic regression modelling approach to evaluate associations between Nugent score category and change in total and/or free 25(OH)D from study visit 1 to study visit 2 as the primary predictor variables. Statistical analyses were conducted using SPSS v24.0 (Armonk, NY: IBM Corp).

## 3. Results

### 3.1. Characteristics of the Study Population

Among the 137 women who met criteria for inclusion in this study, 54 (39%) had a Nugent score indicative of normal flora upon study enrollment (Visit 1), whereas 26 (19%) had intermediate flora, and 57 (42%) had BV. Characteristics of participating women overall and according to baseline Nugent score category are given in Table 1. The percentage of women with Medicaid and with high school or less education varied significantly by Nugent score category. A substantial percentage of women were diagnosed with infection by their prenatal care provider before or between the study visits, with clinically diagnosed BV (diagnosed by Amsel criteria in the clinic setting) and urinary tract infection being the most common at 28% and 26%, respectively. While there was some variation in the percentage of women diagnosed with infection by Nugent score category, there were not significant differences in the percentage across categories. Overall, 37 (27%) participating women had adequate vitamin D status at baseline, whereas 70 (51%) had insufficient vitamin D and 30 (22%) were vitamin D deficient; there were not significant differences in the proportion of women with adequate, insufficient, or deficient vitamin D (based on measured serum 25(OH)D concentration at baseline) according to Nugent score category.

### 3.2. Correlations between Total and Free 25(OH)D in Early and Later Pregnancy

The concentrations of total 25(OH)D and free 25(OH)D were significantly positively correlated at Visit 1 (Spearman coefficient = 0.48, p = 0.001) and Visit 2 (Spearman coefficient = 0.55, p = 0.001). The concentrations of total 25(OH)D at Visit 1 and at Visit 2 were also significantly positively correlated (Spearman coefficient = 0.58, p = 0.001) as were concentrations of free 25(OH)D at Visit 1 and Visit 2 (Spearman coefficient = 0.61, p = 0.001). In paired analysis, there was a significant increase (+3.9 ng/mL) in the median concentration of total 25(OH)D from Visit 1 to Visit 2 (p=0.001) and a significant increase (+0.25 ng/mL) in the median concentration of free 25(OH)D from Visit 1 to Visit 2 (p=0.03). As expected, the vitamin D status (categorized as adequate, insufficient, or deficient) was significantly related to vitamin D status at Visit 2 (p=0.02 for McNemar's test), however, substantial proportions of women shifted status categories from Visit 1 to Visit 2 (Table 2). A majority (53%) of women who were in the deficient category at Visit 1 shifted into the insufficient category at Visit 2; in contrast, 76% of women who were in the adequate category at Visit 1 remained in the adequate category at Visit 2.

### 3.3. Correlations between Total and Free 25(OH)D and Nugent Score

The concentrations of total 25(OH)D at Visit 1 and Visit 2 were significantly negatively correlated with continuous Nugent score at Visit 1 and Visit 2, respectively (Table 3), such that higher levels of total 25(OH)D were associated with lower Nugent score. While the coefficients for the correlation between free 25(OH)D and Nugent score were negative at each visit, no correlations were significant. The change in concentration in total 25(OH)D and free 25(OH)D from Visit 1 to Visit 2 were both significantly negatively correlated with Nugent score at Visit 2 such that an increase in total or free 25(OH)D from Visit 1 to Visit 2 was associated with a lower Nugent score at Visit 2.

### 3.4. Mean and Range of Total and Free 25(OH)D by Nugent Score Categories

**Figure 1:**
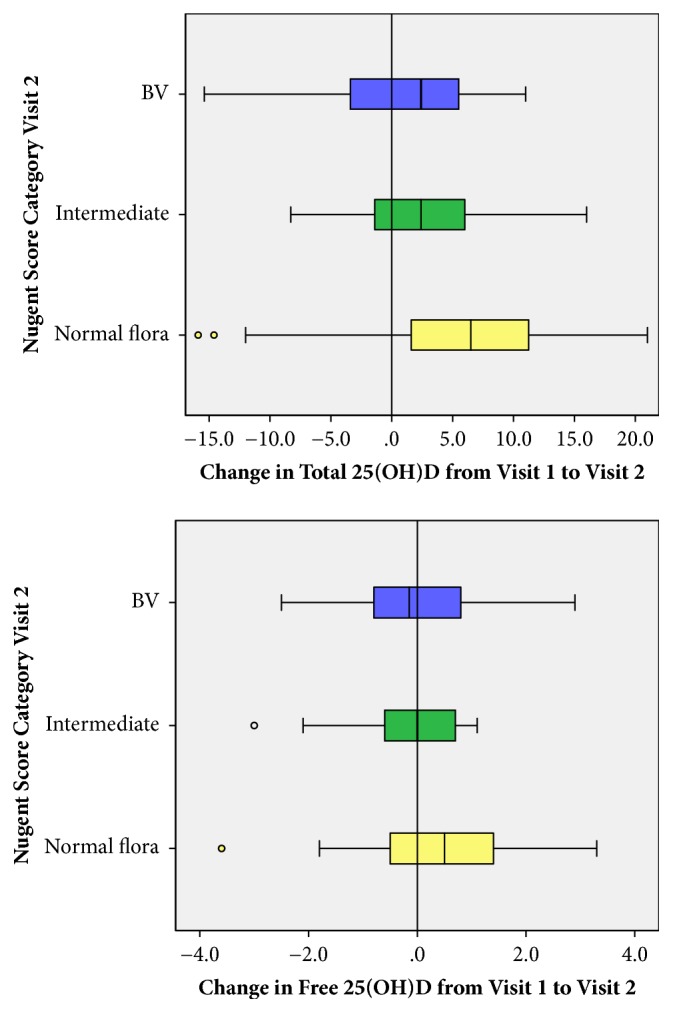
Change in Total and Free 25(OH)D from Visit 1 to Visit 2 by Nugent Category at Visit 2.

The median, interquartile range (IQR), and range for total and free 25(OH)D concentrations for women according to their Nugent score category at Visit 1 and 2 are shown in Table 4. At Visit 1, there were significant differences in the distribution of total 25(OH)D, but not free 25(OH)D according to Nugent score category. At Visit 2, there were not significant differences in the distribution of total or free 25(OH)D across the Nugent score categories; however, the mean change in total 25(OH)D from Visit 1 to Visit 2 (Figure 1) was significantly associated with Nugent score category at Visit 2 with a higher median change for those with normal flora relative to those with intermediate flora or BV. A similar trend was observed with change in free 25(OH)D and Nugent score category at Visit 2, however, the difference in distribution of change in free 25(OH)D did not achieve statistical significant (p = 0.07).

### 3.5. Multinomial Logistic Regression Modelling of Nugent Score Category

The addition of the covariates to the model that contained only the intercept significantly improved the fit between the data and the model that evaluated total vitamin D status and Nugent score category at Visit 1 and Visit 2 (*χ*^2^ = 25, p=0.035) and change in total vitamin D and Nugent score at Visit 2 (*χ*^2^ = 76.2, p<0.001). The addition of the covariates to the model that contained only the intercept significantly improved the fit between the data and the model that evaluated change in free vitamin D and Nugent score at Visit 2 (*χ*^2^ = 59.4, p<0.001) but did significantly improve the fit for the model that evaluated free vitamin D status and Nugent score category at Visit 1 and Visit 2 (*χ*^2^ = 22, p=0.079). The addition of interaction terms did not improve fit statistics.

As shown in Table 5, significant contributions to the models were made by including measures of vitamin D status at Visit 2. At Visit 1, neither total or free 25(OH)D were significantly associated with Nugent score category in the adjusted model. At Visit 2, total 25(OH)D concentration was significantly inversely associated with the risk of BV, such that with an unit increase in total 25(OH) the likelihood of BV decreased by 6% compared to normal flora (aOR:0.94; 95% CI: (0.89, 0.99)). At Visit 2, the change in total 25(OH)D was significantly inversely associated with the risk of BV (aOR: 0.86; 95% CI: (0.78, 0.95)), as was the change in free 25(OH)D (aOR:0.62; 95% CI: (0.41, 0.95) compared to normal flora. In contrast, only the change in total 25(OH)D concentration from Visit 1 to Visit 2 was significantly inversely associated with the likelihood of intermediate compared to normal flora (aOR: 0.90; 95% CI: (0.83, 0.97)).

## 4. Discussion

To our knowledge, this is the first published study that investigates the association between maternal free 25(OH)D and BV occurrence in pregnancy and the Nugent score category of intermediate flora in relation to 25(OH)D status; thus, there is not an established literature to which we can compare our findings on these points. However, our findings supporting that total 25(OH)D is related to the occurrence of BV in pregnancy are consistent with the literature. For example, an earlier US cohort study that examined the association between vitamin D status and BV in pregnancy found a dose-response relationship between the mean serum 25(OH)D concentration and the prevalence of BV in early pregnancy [15]. Despite our findings of an association between serum 25(OH)D and BV, the present study did not find an association between frank vitamin D deficiency (defined as serum total 25(OH)D < 20 ng/mL) and the occurrence of BV. A cohort of Zimbabwean pregnant women also failed to find an association with vitamin D deficiency and BV [35]. In contrast, a cross-sectional study of 440 US pregnant, selected to be representative of the racial-ethnic composition of women of reproductive age in the US, did find that total 25(OH)D < 30 ng/mL (a category combining vitamin D insufficiency and vitamin D deficiency) significantly increased the odds of BV [36]. This disparate findings suggest that large sample sizes may be required to find a significant effect using categorical cut-offs for vitamin D status or that the cut-off values for having a clinically important effect vary by race/ethnicity.

The study of Zimbabwean pregnant women was the first to examine the association between time-varying vitamin D and BV using cohort data, finding that among women who did not have BV at enrollment, deficient or insufficient vitamin D was not associated with incident BV during follow-up [35]. The present study did find an association with change in 25(OH)D from early to later pregnancy, as a continuous measure but not as a categorical measure, and BV later in pregnancy adjusting for baseline Nugent score and 25(OH)D. Further research is needed to confirm this association in other cohorts with measurement of measures of 25(OH)D at more than a single time point in pregnancy and to understand clinical and behavioural factors that may drive changes in vitamin D concentrations during pregnancy.

In addition to including an evaluation of both free and total 25(OH)D and BV in pregnancy, this study has other strengths. BV was diagnosed according to the microbiologic gold standard, Gram staining by Nugent criteria, and the Gram staining was conducted in an established clinical microbiology laboratory with blinding to the identification and clinical status of the participant. Also, the vaginal samples for Gram staining and the serum samples for determination of 25(OH)D status were obtained at the same clinical time points (8-14 weeks' and 24-30 weeks' gestation). A limitation of the present study is its relatively small sample size; a larger sample size may improve model fit, especially for the free vitamin D data, and may improve the ability to find true associations in the data. Another limitation of the present study is the that other nutrient measurements were not available for inclusion in the models. Previous research has established that folate deficiency (as measured by serum folate concentration) is linked with BV status, and that increased dietary intake of other nutrients, including folate, vitamin E, and calcium may decrease the risk of BV [37]. As such, there is the possibility of residual confounding between vitamin D status and other nutrient status contributing to our findings. Future research should focus upon the assessment of overall dietary patterns as well as micronutrient status and their combined effect in shaping the vaginal microbial flora and the occurrence of BV in pregnancy. Another limitation is that, in this study, we could not distinguish persistent vs. recurrent BV between the two study visits as BV was assessed via the gold standard of Gram stain only at the two study visits.

This study did not find stronger associations with free 25(OH)D compared to total 25(OH)D, which may be related to the transient nature of the free 25(OH)D, making it difficult to capture its pivotal level of bioavailability during the sample collection points. To better understand the relationship between vitamin D status and BV, particularly in pregnant women, future studies may need to include other measures that vary during pregnancy and across racial/ethnic backgrounds such as VDBP concentration and other vitamin D metabolites such as the active form 1,25(OH)_2_D as well as polymorphisms of VDBP that may impact 25(OH)D binding. There is evidence that levels of 25(OH)D can vary depending on VDBP genotype that commonly group based on racial/ethnic background [38], which may be an important consideration for black women. Further, the understanding of the relationship between vitamin D status and BV may also require the consideration of other measures of the vaginal microbial composition, including the vaginal microbiome, or intermediate outcomes in the development of BV, such as antimicrobial peptide levels [39]. Given the findings that the change in vitamin D status from early to later pregnancy is important for the occurrence of BV in pregnancy, further research should also focus on identifying the factors that drive the change in 25(OH)D concentrations from early to later pregnancy in black women, including diet and supplementation as well as seasonality, as has been done for cohorts of primarily white women [40].

Finally, despite finding associations between vitamin D status and BV in pregnancy, the effects of vitamin D supplementation on decreasing risk of BV are not supported by the existing literature. Two systematic reviews and meta-analyses of observational studies support that lower vitamin D intake and/or low 25(OH)D levels are associated with an increased risk for BV in pregnancy [41, 42]. Yet randomized trials summarized in the same systematic review do not support a reduction in BV occurrence or recurrence with vitamin D supplementation [42]. In addition, a more recent randomized, placebo-controlled, double-blinded trial of 118 women with symptomatic BV, most of whom were black, found that while those in the antibiotic therapy plus vitamin D supplementation group achieved higher levels of serum 25(OH)D than did those in the antibiotic therapy with placebo group, the increase was not associated with decreased BV recurrence [43]. Thus, future research should also focus on understanding the complex relationships underlying vitamin D status, vitamin D supplementation and the occurrence, recurrence, and persistence of BV in pregnancy.

## 5. Conclusions

In this study of pregnant US black women, vitamin D deficiency and insufficiency were common in early pregnancy (22% and 51%, respectively). Although rates of vitamin D deficiency and insufficiency improved in later pregnancy, with most of the cohort experiencing a mean increase in total 25(OH)D from early to later pregnancy, vitamin D status did worsen for some women in the cohort. The odds of BV later in pregnancy was significantly higher for women who experienced a smaller rise in total 25(OH)D and in those who experienced a smaller rise in free 25(OH)D. Specifically, in multinomial logistic regression modelling controlling for age, parity, prenatal health insurance status, first prenatal BMI, gestational age of sampling, and baseline 25(OH)D and Nugent score, women who experienced a small increase in either total or free 25(OH)D from 8-14 weeks through 24-30 weeks gestation were significantly more likely to have a Nugent score indicative of BV compared with normal flora; a smaller increase in total 25(OH)D over pregnancy was also associated with an increased risk of intermediate flora compared with normal flora. Further research is needed to examine for change in vitamin D status over the course of pregnancy and the occurrence of BV and other measures of vaginal microbial composition and to identify factors that influence change in vitamin D status over pregnancy.

## Figures and Tables

**Table 1:** Characteristics of participating women, overall and categorized by baseline nugent score.

**Characteristic**	**Overall ** **N = 137**	**Baseline Nugent Score Category**			***p*-value**
		**Normal**	**Intermediate**	**BV**	
		**n = 54**	**n = 26**	**n = 57**	
Age in years, mean (sd)	24.3 (4.3)	25.3 (4.7)	23.5 (3.8)	23.7 (4.1)	0.09

No prior birth, n (%)	70 (51%)	26 (48%)	16 (62%)	28 (49%)	0.52

Married or cohabiting, n (%)	64 (47%)	21 (39%)	14 (54%)	29 (51%)	0.32

Medicaid insurance, n (%)	***102 (75***%**)**	***33 (61***%**)**	***23 (89***%**)**	***46 (81***%**)**	***0.01***

≤12th grade education, n (%)	***69 (50***%**)**	***20 (37***%**)**	***14 (54***%**)**	***35 (61***%**)**	***0.03***

Infection diagnosed before or between visits, n (%)					
BV^1^	38 (28%)	11 (20%)	9 (35%)	18 (32%)	0.28
Chlamydia	16 (12%)	4 (7%)	4 (15%)	8 (14%)	0.45
Gonorrhea	3 (2%)	1 (2%)	1 (4%)	1 (2%)	0.81
Trichomoniasis	12 (9%)	4 (7%)	4 (15%)	4 (7%)	0.46
Urinary tract infection	35 (26%)	11 (21%)	6 (23%)	18 (32%)	0.37
Other bacterial infection	5 (4%)	4 (7%)	1 (4%)	0	0.11

Antibiotic receipt, n (%)					
Before Visit 1	34 (25%)	11 (20%)	11 (42%)	12 (21%)	0.07
Between Visit 1 and 2	37 (27%)	11 (20%)	8 (31%)	18 (32%)	0.37

Total 25(OH)D, n (%)					
Adequate	37 (27%)	17 (31%)	7 (27%)	13 (23%)	0.56
Insufficient	70 (51%)	30 (56%)	12 (46%)	28 (49%)	
Deficient	30 (22%)	8 (14%)	6 (23%)	16 (28%)	

***Bold, italic font,*** indicates a significant difference for women according to Nugent score category at *α* = 0.05.

^1^ Diagnosed per Amsel's criteria as part of prenatal care encounters.

**Table 2:** Vitamin D Status at Visit 1 and Visit 2.

**Vitamin D Status at Visit 1**	**Vitamin D Status at Visit 2**		
	**Adequate**	**Insufficient**	**Deficient**
	n (row%)	n (row%)	n (row%)
Adequate, n = 37	28 (76%)	8 (22%)	1 (2%)
Insufficient, n = 70	26 (37%)	35 (50%)	9 (13%)
Deficient, n = 30	4 (14%)	16 (53%)	10 (33%)

Total = 137	N = 58 (42%)	N = 59 (43%)	N = 20 (15%)

**Table 3:** Correlations between measures of 25(OH)D and Nugent Score at study visits.

**Vitamin D Measure (ng/mL)**	**Spearman Correlation Coefficient (p-value) with Nugent Score at Visit**	
	**Visit 1**	**Visit 2**
**Visit 1**		
Total 25(OH)D	***-0.23 (0.007)***	+ 1.20 (0.17)
Free 25(OH)D	-0.05 (0.59)	+ 0.15 (0.09)
**Visit 2**		
Total 25(OH)D	-	***-0.16 (0.04)***
Free 25(OH)D	-	-0.03 (0.70)
**Change Visit 1 to Visit 2**		
Total 25(OH)D	-	***- 0.30 (0.001)***
Free 25(OH)D	-	***- 0.18 (0.04)***

***Bold, italic font,*** indicates a significant correlation at *α* = 0.05.

**Table 4:** Comparison of total and free 25-OH-D by Nugent Score category at study visits.

**Vitamin D ** **Measure (ng/mL)**	**Nugent Score Category**			**p-value for one-way ANOVA of group means**
	**Normal Flora**	**Intermediate Flora**	**BV**	
	**n = 54**	**n = 26**	**n = 57**	
**Visit 1**				
Total 25(OH)D				
Median (IQR)	***27.7 (10.0)***	***23.7 (12.7)***	***23.3 (10.1)***	***0.02***
Min, Max	15.7, 40.9	10.4, 44.5	13.1, 43.7	
Free 25(OH)D				
Median (IQR)	3.8 (1.6)	4.1 (1.3)	3.7 (1.8)	0.75
Min, Max	1.4, 7.9	2.0, 6.3	1.4, 8.9	
**Visit 2**				
Total 25(OH)D				
Median (IQR)	31.2 (13.4)	28.2 (13.5)	26.6 (9.9)	0.30
Min, Max	14.8, 53.9	14.4, 45.6	11.5, 44.6	
Free 25(OH)D				
Median (IQR)	4.0 (2.9)	4.0 (2.5)	3.9 (2.2)	0.69
Min, Max	1.2, 12.9	1.2, 8.3	1.5, 9.1	
**Visit 2-Visit 1**				
Δ Total 25(OH)D^1^				
Median (IQR)	***5.0 (10.2)***	***2.4 (10.8)***	***1.2 (9.4)***	***0.003***
Min, Max	-14.6, 5.0	-15.9, 18.0	-15.4, 11.0	
Δ Free 25(OH)D^1^				
Median (IQR)	0.40 (1.5)	0.01 (1.9)	0.01 (1.6)	0.07
Min, Max	-2.1, 5.0	-3.6, 2.7	-3.0, 5.8	

***Bold, italic font,*** indicates a significant difference for women according to Nugent score category at *α* = 0.05.

^1^ Change in 25(OH)D concentration from Visit 1 to Visit 2 (concentration at Visit 2—concentration at Visit 1).

**Table 5:** Multinomial odds of Nugent score category (intermediate flora and bv relative to normal flora) by total and free 25(oh)d concentration.

**Vitamin D Measure (ng/mL)**	**Nugent Score Category**			
	**Intermediate Flora**		**BV**	
	**Coefficient**	**aOR (95**%** CI)**	**Coefficient**	**aOR (95**%** CI)**
**Visit 1** ^**1**^				
Total 25(OH)D	-0.04	0.96 (0.89. 1.03)	-0.04	0.96 (0.91, 1.01)
Free 25(OH)D	0.10	1.11 (0.78, 1.58)	0.06	1.06 (0.80, 1.41)
**Visit 2** ^**1**^				
Total 25(OH)D	-0.03	0.97 (0.92, 1.02)	-0.06	***0.94 (0.89, 0.99)***
Free 25(OH)D	-0.18	0.84 (0.63, 1.11)	-0.13	0.88 (0.69, 1.10)
**Visit 2-Visit 1** ^**2**^				
Δ Total 25(OH)D	-0.11	***0.90 (0.83, 0.97)***	-0.15	***0.86 (0.78, 0.95)***
Δ Free 25(OH)D	-0.31	1.1 (0.78, 1.56)	-0.47	***0.62 (0.41, 0.95)***

***Bold, italic font,*** indicates a significant difference for women according to Nugent score category at *α* = 0.05.

^1^Model adjusts for maternal age, parity, insurance status (Medicaid, private), first prenatal BMI, gestational age of visit, and receipt of antibiotics in the month prior to the visit.

^2^Model adjusts for maternal age, parity, insurance status (Medicaid, private), and first prenatal BMI; baseline 25(OH)D concentration, Nugent score, and gestational age of visit, in addition to number of weeks between visits and receipt of antibiotics between visits.

## Data Availability

Readers may contact the lead author, Dr. Anne L. Dunlop, at amlang@emory.edu to request access to the deidentified data used for the study described herein.
